# The use of an active learning approach in a SCALE-UP learning space improves academic performance in undergraduate General Biology

**DOI:** 10.1371/journal.pone.0197916

**Published:** 2018-05-24

**Authors:** Gokhan Hacisalihoglu, Desmond Stephens, Lewis Johnson, Maurice Edington

**Affiliations:** 1 Biological Sciences Department, Florida A&M University, Tallahassee, Florida, United States of America; 2 Teaching & Learning Center and Department of Mathematics, Florida A&M University, Tallahassee, Florida, United States of America; 3 Department of Physics, Florida A&M University, Tallahassee, Florida, United States of America; 4 Department of Chemistry, Florida A&M University, Tallahassee, Florida, United States of America; TNO, NETHERLANDS

## Abstract

Active learning is a pedagogical approach that involves students engaging in collaborative learning, which enables them to take more responsibility for their learning and improve their critical thinking skills. While prior research examined student performance at majority universities, this study focuses on specifically Historically Black Colleges and Universities (HBCUs) for the first time. Here we present work that focuses on the impact of active learning interventions at Florida A&M University, where we measured the impact of active learning strategies coupled with a SCALE-UP (Student Centered Active Learning Environment with Upside-down Pedagogies) learning environment on student success in General Biology. In biology sections where active learning techniques were employed, students watched online videos and completed specific activities before class covering information previously presented in a traditional lecture format. In-class activities were then carefully planned to reinforce critical concepts and enhance critical thinking skills through active learning techniques such as the one-minute paper, think-pair-share, and the utilization of clickers. Students in the active learning and control groups covered the same topics, took the same summative examinations and completed identical homework sets. In addition, the same instructor taught all of the sections included in this study. Testing demonstrated that these interventions increased learning gains by as much as 16%, and students reported an increase in their positive perceptions of active learning and biology. Overall, our results suggest that active learning approaches coupled with the SCALE-UP environment may provide an added opportunity for student success when compared with the standard modes of instruction in General Biology.

## Introduction

Over the past several decades, there has been a substantial increase in research focusing on increasing student engagement, retention and critical thinking skills, especially in STEM fields [1]. Much of this research has centered around Classroom Assessment Techniques developed by Angelo and Cross [2], which provide college faculty with systematic ways to measure learning in the classroom. Building on these tools, Bergmann and Sams developed a flipped teaching pedagogy for chemistry students at Woodland Park High School in Colorado that facilitated learning for rural students who had long absences from school due to distances traveled to and from school and to extracurricular activities [3]. In this model, students watched video lectures prior to returning to class, followed by engaging in well-designed active learning activities in class. The use of video lectures was useful, as the instructors no longer had to spend their time prepping for lecture in order to introduce foundational information, but instead could build on these concepts, freeing up class time for more challenging concepts.

There is convincing evidence that the flipped classroom model can improve student performance. A study of Integrated Pharmacotherapy I at Texas A&M Health Center found that post-test mean scores, final grades, and student satisfaction were improved in flipped courses compared to courses primarily taught through a traditional lecture format [4]. After examining 250 studies, Freeman et al. (2014) [1] concluded that active learning increased student exam scores in STEM courses by as much as 6%. In an upper-level Physical Chemistry course, Gross et al. (2015) [5] reported a 12% improvement on exam scores and additional gains, especially for underperforming students, in a flipped classroom. One University of Minnesota study by Cotner et al. (2013) [6] described improved student performance that exceeded the expectations associated with active learning classrooms in introductory biology. A recent Iowa State University study by Rands and Gansemer-Topf (2017) [7] showed that classroom design helped students form learning communities with an optimal level of challenge and enhanced student engagement. In addition, Stolzfus and Libarkin (2016) [8] and Soneral and Wyse (2017) [9] have both reported that the key to success was the active learning itself, and therefore could be achieved in low-tech classrooms as well.

**Fig 1:**
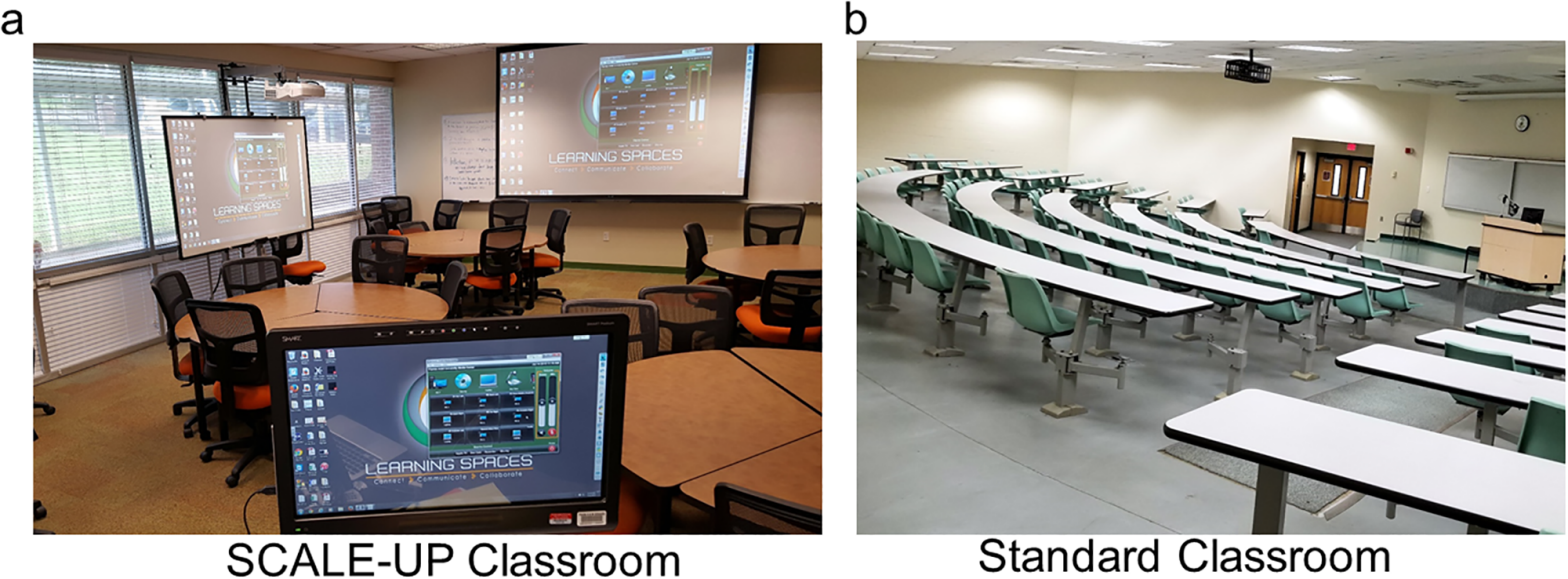
Views of two types of classrooms used in this study. A) SCALE-UP classroom with round tables, moveable chairs, and smart boards; and B) standard auditorium classroom with fixed rows of seats.

The primary benefit of Bergmann and Sams’s flipped classroom approach was that students were able to work at their own pace and engage with the most challenging material in the presence of an expert: their instructor. Robert Beichner at North Carolina State University combined the work of Angelo and Cross and Bergmann and Sams by adding specific technological and social interaction enhancements in the classroom, in an environment termed SCALE-UP (Student-Centered Active Learning Environment with Upside-down Pedagogies) [10]. In a SCALE-UP classroom, students are assigned to groups of three at round tables that seat nine students. Each table contains three computers (one for each group), and there are whiteboards and/or easels strategically placed around the room. After students have completed before-class activities, they are assigned active learning and ponderable activities that have been shown to improve their overall understanding of the material while improving their critical thinking skills. In these classrooms, all aspects of the environment are controlled, from the size and constitution of the groups, to the table diameters and length of the course [10]. A particular advantage of SCALE-UP comes from this unique classroom layout that helps students foster collaborations, and increases student-to-student and student-to-teacher interactions while increasing overall student engagement as compared to a traditional lecture hall (Fig 1).

Several studies have reported the effectiveness of active learning [1, 3, 4, and 5]; however, there is not any published research on these approaches with HBCU students. In particular, whether active learning in a SCALE-UP environment results in similarly increased outcomes and enhanced student learning in STEM at HBCUs is unknown.

In this study, we implemented an active learning curriculum coupled with the flipped teaching methodology and an infusion of educational technology in a SCALE-UP environment at Florida A&M University, which is an HBCU. In the General Biology I course at Florida A&M University, the flipped classroom teaching method and SCALE-UP environment were merged to promote active learning. Most of the information normally disseminated through a traditional lecture was delivered via online videos coupled with reading activities to be done before class, freeing time for group learning activities during the class.

In addition to the use of online videos, both student response systems (clickers) and personal Wi-Fi-enabled devices were also used in class. The clickers and Wi-Fi devices provided students with a way to receive instant formative feedback and provided the instructor with an opportunity to shift the instructional emphasis as needed based upon where students in the class were on a particular topic. Students were given the opportunity to utilize their own devices to take advantage of the findings of Katz et al. (2017) [11], which indicated that students preferred using personal devices over clickers.

The objectives of this study were (1) to describe the experience of implementing an active learning curriculum using the flipped teaching methodology in a SCALE-UP environment at an HBCU from an instructor’s perspective, and (2) to assess the effectiveness of the implementation on student learning in General Biology I at Florida A&M University (FAMU).

## Materials and methods

### Course structure

This study was carried out over the course of three semesters using SCALE-UP compared to six semesters of a standard freshman General Biology I course. This is a required course for all STEM and health science majors covering molecular and cellular biology. Table 1 summarizes the general characteristics of students in the SCALE-UP model (3) and standard course sections. Class population demographics were similar for all sections and consistent with the university demographics (approximately 90% African-Americans, 6% Caucasians, and 4% Hispanics, Asians, and others). Furthermore, FAMU is a public HBCU with an 83% freshman retention rate, which is in the top 25% of all four-year institutions.

**Table 1:** Summary of demographic info of students who attended the introductory biology courses under the study.

Semester	Class type	Class size	% Female	% Male	Class time (min/week)	Major
Fall 2016	SCALE-UP	48	83	17	150	Pharmacy (28%), Biology/PreMed (20%), Allied Health (20%), PreNursing (16%), Others (16%)
Spring 2016	SCALE-UP	33	79	21	150	Pharmacy (5%), Biology/PreMed (41%), Allied Health (25%), Social Sci. (23%), Others (24%)
Fall 2015	SCALE-UP	57	78	22	150	Pharmacy (10%), Biology/PreMed (20%), Allied Health (24%), Social Sci. (9%), Others (16%)
Spring 2015	Standard	39	64	36	150	Pharmacy (8%), Biology/PreMed (21%), Allied Health (27%), Social Sci. (31%), Others (13%)
Spring 2014	Standard	42	74	26	150	
Fall 2013	Standard	63	83	17	150	Pharmacy (26%), Biology/PreMed (29%), Allied Health (28%), PreNursing (4%), Others (13%)
Spring 2013	Standard	46	79	21	150	Social Sci. (29%), Biology/PreMed (29%), Allied Health (20%), Business (8%), Others (14%)
Fall 2011	Standard	100	70	30	150	Pharmacy (25%), Biology/PreMed (38%), Allied Health (18%), Others (19%)
Spring 2011	Standard	77	74	26	150	Allied Health (30%), Biology/PreMed (19%), Gen. Studies (9%), Agriculture (8%), Others (34%)

The SCALE-UP course sections were taught in an active learning classroom space with round tables, multiple projectors, smart boards, and clickers. Student enrollment in these sections was capped at 42 to 60 students, depending on the classroom seating capacity (Fig 1a). The standard course sections were in a standard university lecture room (Fig 1b) with a student enrollment of up to 100 students. On average SCALE-UP sections consisted of 80% female and 20% male students; standard sections consisted of 74% females and 26% males (Table 1). These student distributions were consistent with percentages for students enrollment in the Biology Department at FAMU (77% female and 23% male students). All nine semesters of the course were taught by the same instructor, with the same chapter and homework assignment coverage [12], and all SCALE-UP sections had the same in-class activities and clicker quizzes. In standard lecture classes, students were assigned readings prior to lecture and homework afterward to reinforce the lectures.

**Fig 2:**
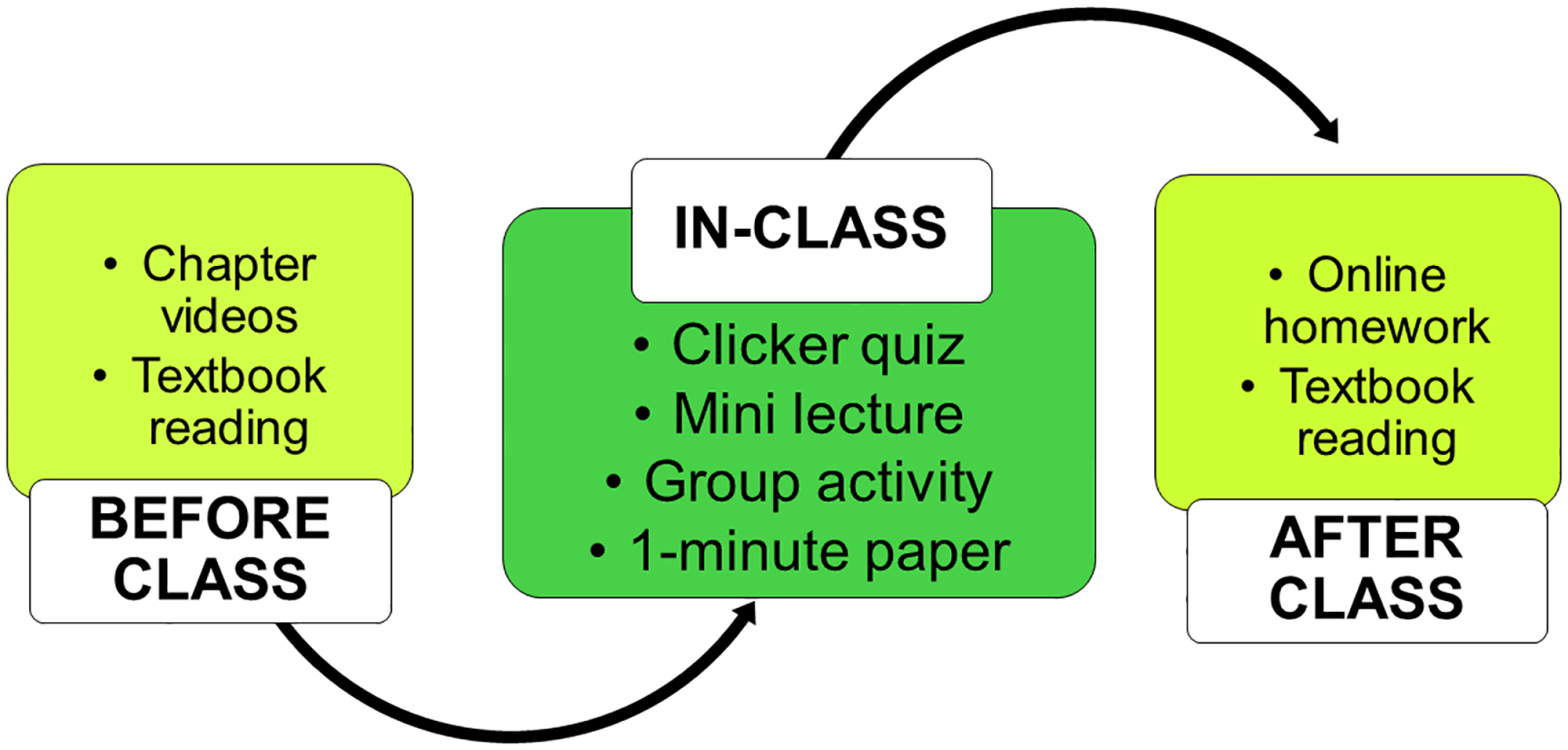
A representation of the SCALE-UP classroom model flowchart used in this study.

All sections met 150 minutes per week for a 15-week semester. The SCALE-UP format sections consisted of clicker quizzes, mini-lectures, and group activities (Fig 2) while the traditional sections were taught using a standard lecture format with slide presentations. The students in the SCALE-UP-based course sections had one of several active learning techniques incorporated into in-class time weekly (Table 2).

**Table 2:** Examples of ponderable and group active learning exercises used in SCALE-UP course sections of General Biology I.

**Ponderable Activities**:
GIANT PLANT CELL: Every student is assigned a number. When the instructor mentions a specific organelle, s/he calls a random number and then the student with that number draws the organelle on the white board. ILLUSTRATED STORY OF PHOTOSYNTHESIS: Every student works on creating a story based on C3 plant photosynthesis that includes the following keywords: ATP, water, oxygen, ATP-synthase, thylakoid membrane, glucose, C3 sugars, sunlight, NADPH, and NADP^+^. CELL DIVISION: Every student compares and contrasts meiosis with mitosis, discussing their stages, similarities, and differences.
**Group Active Learning Exercises**:
CONCEPT MAPPING: Each table group is asked to construct a concept map of a specific chapter by beginning with a main concept and branching out to specific topics. One student per group explains it to the class. MODEL CELLULAR RESPIRATION: Each table group is asked to model cellular respiration using their notes, including the major molecules, enzymes, and number of ATPs. THINK-PAIR-SHARE: Each table group is asked to think about the answer to a given specific question, pair with another group, debate their answers, and share it with whole class. 1-MINUTE PAPER: Each table group is asked to write a sixty second summary of their understanding of three to five major points and their significance.

SCALE-UP sections consisted of mini-lectures that were approximately 15 minutes in length and follow-up discussion. Students were then assigned to specific tables and groups, with group and table assignments re-shuffling periodically throughout the semester. Pre-class assignments consisted of reading from the textbook and watching of 10–12 min online videos related to the specific topic covered, and post-class homework was assigned following the section.

### Ethics statement

The Institutional Review Board at Florida Agricultural and Mechanical University has approved the work described in this study under “FAMU IRB Approval Number 013–28”.

### Pre- and post-test

The summative assessments for all semesters consisted of four major written exams with similar content coverage and question formats. A departmental 10-item pre-test and post-test was used to measure student gains in both the standard and SCALE-UP sections studied. The items were adapted from departmental in house pre- and post-tests, and reviewed for content validity by researchers with expertise in undergraduate teaching, assessment, and SCALE-UP active learning practices. Pre- and post-tests cover General Biology I subject matter and typically were assigned on the first and last day of classes.

### Student surveys

**Fig 3:**
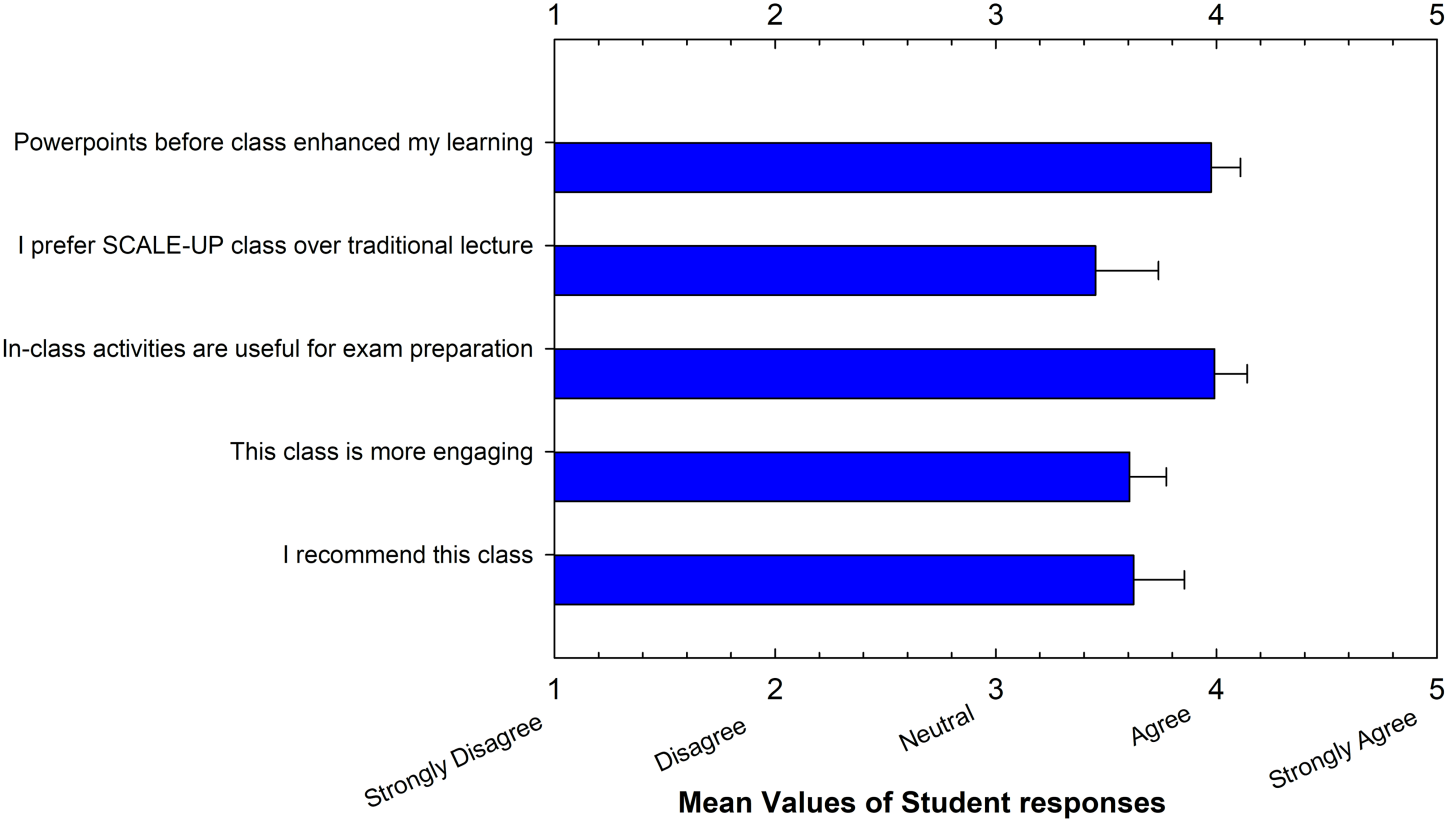
Student experiences survey and mean scores of each survey item with standard errors.

An online exit survey was used to analyze the student perception of the SCALE-UP format (Table 3, Fig 3). The survey consisted of six statements with answer options ranging from 1 to 5 (1 = Strongly Disagree, 2 = Disagree, 3 = Neutral, 4 = Agree, and 5 = Strongly Agree) as well as open-ended questions with no time restriction. The survey items were adapted from departmental surveys, and reviewed for content validity by researchers with expertise in undergraduate teaching, assessment, and SCALE-UP active learning practices. The survey results were analyzed based on the mean scores of each survey item with standard errors of introductory biology courses studied. The response rate for the survey was approximately 81 percent of the students enrolled. The survey also included an open-response question in which students were asked to explain which aspects of the course they liked or disliked. The open-ended responses were read and grouped into five theme categories by one co-author. A second co-author independently scored the responses into categories. Additionally, the theme categories were discussed between investigators to ascertain the quality of the findings. The interrater reliability was approximately 93 percent agreement.

**Table 3:** Perceptions of students enrolled in SCALE-UP General Biology I course sections.

**Student Survey Responses to “Why the SCALE-UP format was beneficial?”**
***Increased engagement due to working with peers and group activities*** “Being able to get help from tablemates when someone doesn't understand something.” “I liked that we get to do activities and get credit for doing them.” “I liked in class activities and the way Dr. H teaches.” ***Access to study resources before and after class*** “I liked how you must read and study the content before you come to class and then you go over the info when you get to class the next day.” “I liked the videos on Blackboard. If I did not understand something from the text book, I had another resource to help me learn the material.” “I liked reviewing lecture before and after the class.” ***1-minute paper summaries*, *Drawing boards*, *clickers*** “Clicker quizzes because you get your results immediately and know exactly what you need to study as opposed to a traditional paper quiz.” “I liked the fact that we did a 1-minute summary of what we learned at the end of class which made me think back on the material I just learned.” “I liked to connect certain terms by drawing diagrams. It really helped my understanding.”
**Student Survey Responses to “The Disadvantages and challenges of SCALE-UP”**
***Preferred not working in groups*** “Not my type of learning.” “Some people prefer working alone.” “Group work is not for me.” ***Preferred standard lecture classes*** “I prefer a traditional lecture class.” “SCALE-UP class wasn’t a great idea.”

### Outcome measures

As a measure of student outcomes, students’ final course averages were used for success rates in this study.

### Data analysis

All analysis was completed using IBM SPSS Statistics 22 software (SPSS, Chicago, IL). One-way analysis of variance (ANOVA) was used to compare group differences by semesters, with post hoc analysis conducted at p<0.05 level where appropriate. Relationships between variables were examined using linear regression.

## Results

### Course general characteristics

Table 1 summarizes the demographic information for the nine semesters of General Biology I used in this study. A total of 505 students enrolled in the course during the nine semesters studied. Attendance was required as a university rule in all sections. Overall, students were more likely to be biology, pre-med, pharmacy, and allied health majors.

### Student perceptions of the SCALE-UP classes

The active classroom used for SCALE-UP had smart boards, moveable round tables for group activities, clickers, and projectors on all sides. We implemented a SCALE-UP flipped classroom model during the 2015 and 2016 academic years. In general, we found the use of the SCALE-UP model allowed us to cover the same amount of material covered in standard course sections.

The exit survey revealed that students were generally positive about their active learning and SCALE-UP class experience (Fig 3). Students agreed that in-class activities were useful for exam preparation (mean = 3.99) and class was more engaging than standard (mean = 3.77). Students were also agreed that they would recommend the SCALE-UP class (mean = 3.63) (Fig 3).

In addition to quantitative data, we have included qualitative data of student perceptions. The focus group was the entire class of SCALE-UP sections. Five main themes emerged regarding students’ perceptions of the SCALE-UP format (summarized in Table 3). Students who said they liked the SCALE-UP format commented that they enjoyed being in a table group, student–student interaction, access to study resources before and after the class, one-minute summaries, clicker quizzes, and online homework. It should be noted that a small number of students indicated they preferred a traditional lecture format and working alone (Table 3).

### Pre- and post-test

**Fig 4:**
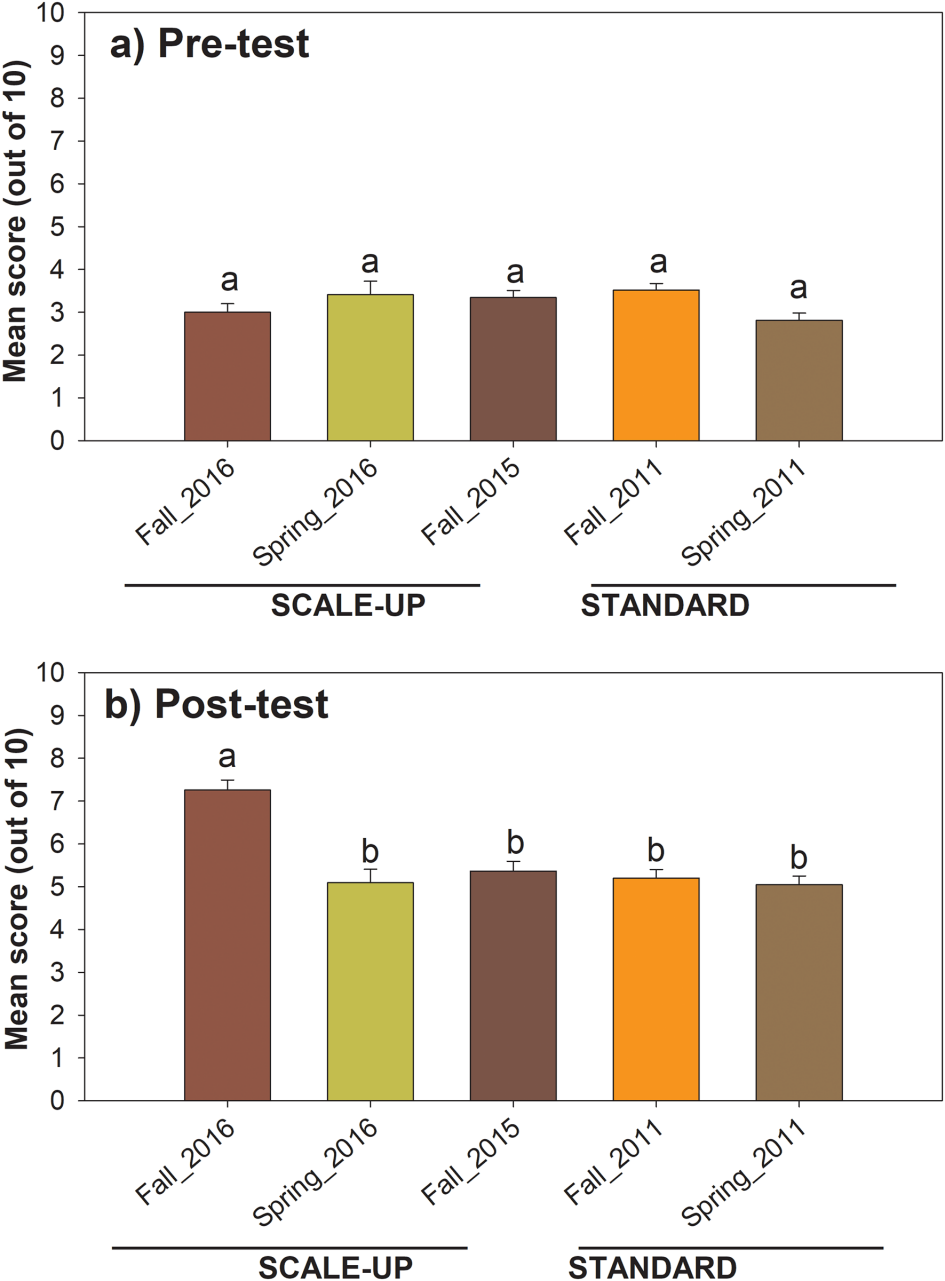
Comparison of pre-test and post-test gain of knowledge in SCALE-UP and standard sections of General Biology I.

To measure student gain of knowledge, a required 10 question pre- and post-test was administered on the first and last day of class, respectively. There was no statistically significant difference in pre-test results between semesters or between SCALE-UP and standard sections (Fig 4). Based on the post-test, the average learning gain was 27% for the SCALE-UP sections and 19% for the standard sections (Fig 4). For the Fall 2016 SCALE-UP section, there was a 43% increase in student learning gain.

### Academic performance and success rates

**Fig 5:**
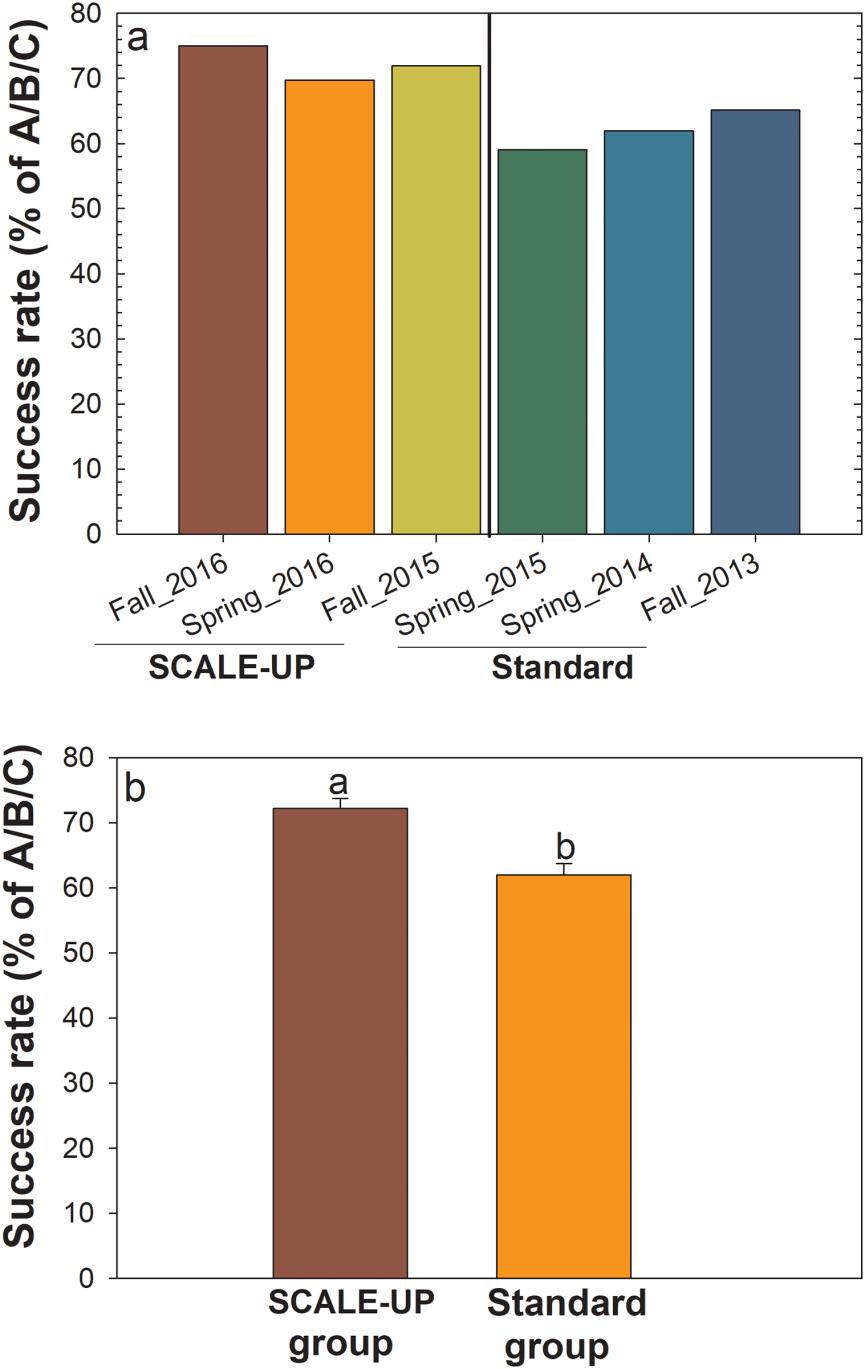
Success rates before (standard) and after (SCALE-UP) in General Biology I. a) individual semesters; and b) averages of SCALE-UP versus standard semesters studied.

Our results revealed that the ABC grade success rates were enhanced in the SCALE-UP model (Fig 5). Overall, there was a 16% increase in passing rates in combined SCALE-UP sections compared with standard sections. Specifically, ABC success rates reached a minimum 75% for Fall 2016 SCALE-UP sections (Fig 5a and 5b).

## Discussion

It cannot be disputed that HBCUs play a critical role in STEM education. The most recent data indicates that 25% of the bachelor’s degrees and 75% of doctoral degrees awarded to African Americans are conferred by HBCUs. Additionally, approximately 65% of the African American physicians and 50% of African Americans engineers earned degrees from HBCUs [13]. Although HBCUs produce a significant number of African American STEM majors, the impact of pedagogical practices at these institutions are not widely represented in the STEM education research literature. Improving six-year graduation rates is one of the major challenges of HBCUs. The current study explored how active learning and SCALE-UP classrooms supported student success at Florida A&M University, an HBCU. It is generally accepted that improving passing rates of major introductory courses can help increasing graduation rates, positively impact retention rates, and therefore impact STEM pipeline [1 and 14].

Here we report the successful design, implementation, and assessment of active learning techniques paired with the SCALE-UP model in an undergraduate General Biology I course at a HBCU. Overall, all course sections had similar demographic distribution concerning majors, year classification, gender, and ethnicity backgrounds (Table 1).

In recent years, FAMU has designed several SCALE-UP style active learning classrooms equipped with innovative technology to better engage students in lower-level STEM courses (Fig 1a). These physical spaces have been upgraded to include round tables that can accommodate two to three groups of three, whiteboards around the room and teacher podiums with smart technology. Faculty teaching in these rooms can incorporate group activities that require presentations and the use of technology with little to no disruption. By contrast, Fig 1b shows the view of a standard model classroom space with single-student tables and chairs in an auditorium. Furthermore, implementing active learning spaces and teaching practices are in alignment with the FAMU’s strategic plan priorities, such as providing exceptional student experiences along with increasing retention and graduation rates [15].

In an effort to better understand the associated academic performance, we compared three SCALE-UP model sections (Fall 2015, Spring 2016, and Fall 2016) with six standard sections (Spring 2011, Fall 2011, Spring 2013, Fall 2013, Spring 2014, and Spring 2015) with the same subject coverage, the same number of summative exams, and identical online homework assignments (Table 1). An exit survey revealed that students felt their learning experience was enhanced and they enjoyed interactive learning in the SCALE-UP model sections (Fig 3), similar to results previously obtained in other STEM courses [1, 5, and 11].

The major findings of this study are that active learning approaches implemented in SCALE-UP spaces were correlated with higher post-test scores, final grades, and passing rates compared with the traditional standard mode of instruction in classrooms (Figs 4 and 5). In addition, students who scored higher on post-tests also had significantly higher final course grades, mirroring previously reported work [16].

Our survey results demonstrated that most students liked the SCALE-UP paired with active learning techniques approach and found this format beneficial due to in-class group activities, online homework, and chapter videos (Table 2, Fig 3). This is consistent with a Texas A&M University study [4], which showed that students responded more positively on course satisfaction surveys when taking flipped and active learning courses than when taking similar courses in a traditional format. The impacts of this approach are encouraging for several reasons. First, the implementation of the SCALE-UP teaching methodology is relatively new in the HBCU environment in general, and at FAMU in particular. FAMU, for example, has a 90% African American enrollment coupled with a 40.7% 6-year graduation rate. The success seen in this work has the potential to impact the way students are taught STEM topics throughout the HBCU community and to dramatically increase the numbers of underrepresented students receiving STEM degrees. The academic gains we observed are consistent with the findings of previous studies in non-HBCU majority institutions [1, 5, 6, and 17]. The time that pre-class activities freed up was used to carry out in-class active learning exercises in SCALE-UP classrooms and helped the SCALE-UP students outperform the standard course sections in General Biology I.

## Conclusions

Using a SCALE-UP design, we introduced an active learning environment to teach General Biology I at FAMU. Overall, our results suggest that the combination of the active learning approach and the SCALE-UP space have had a measurable and positive impact on student learning when compared to traditional modes of instruction at FAMU. To our knowledge, the present study is the first to report such successes using the SCALE-UP active learning approach for a course at an HBCU. Therefore, it serves as a foundation for further studies to promote student engagement with course material, active collaboration, and the ultimate goal of increased student learning. These research results clearly support strategic plan goals to increase the number of students graduating in core STEM fields such as biology [14, 18]. We believe we have developed a model that can be adopted to reform the teaching of STEM courses and therefore, a focus on other undergraduate courses and HBCUs may also be useful for future studies.
